# Concerted Activity of IgG1 Antibodies and IL-4/IL-25-Dependent Effector Cells Trap Helminth Larvae in the Tissues following Vaccination with Defined Secreted Antigens, Providing Sterile Immunity to Challenge Infection

**DOI:** 10.1371/journal.ppat.1004676

**Published:** 2015-03-27

**Authors:** James P. Hewitson, Kara J. Filbey, Julia Esser-von Bieren, Mali Camberis, Christian Schwartz, Janice Murray, Lisa A. Reynolds, Natalie Blair, Elaine Robertson, Yvonne Harcus, Louis Boon, Stanley Ching-Cheng Huang, Lihua Yang, Yizheng Tu, Mark J. Miller, David Voehringer, Graham Le Gros, Nicola Harris, Rick M. Maizels

**Affiliations:** 1 Institute of Immunology and Infection Research, and Centre for Immunity, Infection and Evolution, University of Edinburgh, Edinburgh, United Kingdom; 2 École Polytechnique Fédérale de Lausanne, Lausanne, Switzerland; 3 Malaghan Institute, Wellington, New Zealand; 4 Department of Infection Biology, University Clinic Erlangen, Erlangen, Germany; 5 Bioceros Holding BV, Utrecht, The Netherlands; 6 Department of Pathology and Immunology, Washington University in St. Louis, St. Louis, Missouri, United States of America; 7 Department of Internal Medicine, Washington University in St. Louis, St. Louis, Missouri, United States of America; New York University, UNITED STATES

## Abstract

Over 25% of the world's population are infected with helminth parasites, the majority of which colonise the gastrointestinal tract. However, no vaccine is yet available for human use, and mechanisms of protective immunity remain unclear. In the mouse model of *Heligmosomoides polygyrus* infection, vaccination with excretory-secretory (HES) antigens from adult parasites elicits sterilising immunity. Notably, three purified HES antigens (VAL-1, -2 and -3) are sufficient for effective vaccination. Protection is fully dependent upon specific IgG1 antibodies, but passive transfer confers only partial immunity to infection, indicating that cellular components are also required. Moreover, immune mice show greater cellular infiltration associated with trapping of larvae in the gut wall prior to their maturation. Intra-vital imaging of infected intestinal tissue revealed a four-fold increase in extravasation by LysM^+^GFP^+^ myeloid cells in vaccinated mice, and the massing of these cells around immature larvae. Mice deficient in FcRγ chain or C3 complement component remain fully immune, suggesting that in the presence of antibodies that directly neutralise parasite molecules, the myeloid compartment may attack larvae more quickly and effectively. Immunity to challenge infection was compromised in IL-4Rα- and IL-25-deficient mice, despite levels of specific antibody comparable to immune wild-type controls, while deficiencies in basophils, eosinophils or mast cells or CCR2-dependent inflammatory monocytes did not diminish immunity. Finally, we identify a suite of previously uncharacterised heat-labile vaccine antigens with homologs in human and veterinary parasites that together promote full immunity. Taken together, these data indicate that vaccine-induced immunity to intestinal helminths involves IgG1 antibodies directed against secreted proteins acting in concert with IL-25-dependent Type 2 myeloid effector populations.

## Author Summary

Despite the high prevalence of gastrointestinal helminth parasites in human and animal populations throughout the world, no vaccines are yet available and we lack understanding of how anti-parasite protective immunity may operate effectively. We have used a model system with a natural mouse nematode parasite, *Heligmosomoides polygyrus*, which establishes long-term chronic infection in laboratory mice through the secretion of immunosuppressive molecules. Immunization of mice with as few as 3 secreted proteins, collected from parasites in vitro, confers complete immunity to challenge infection. We show here that immunity requires specific IgG1 antibodies directed to the secreted products, acting together with innate myeloid cells that require activation through the canonical Type 2 cytokine receptor, IL-4Rα, as well as through a pathway not previously known to be involved in effector mechanisms, IL-25. These myeloid cells act to trap and envelop helminth larvae while in the submucosal tissues of the small intestine, massing in large numbers and preventing their maturation and exit into the gut lumen. Thus the combined effects of specific antibodies from the adaptive immune system, and Type 2 cytokine activation of the innate immune system, co-operate to ensure elimination of the helminth parasite.

## Introduction

The immune system has evolved suites of defense mechanisms to protect against infectious pathogens of all types ranging from viral and bacterial micro-organisms to more complex eukaryotic fungi, protozoa and helminths. In contrast to our detailed knowledge of anti-microbial immune mechanisms, however, we have yet to develop a clear picture of how immunity acts to eliminate parasites such as gastrointestinal helminths which even today infect over 1 billion people across the world [1].

The need to understand how the immune system can successfully eliminate helminth parasites is accentuated by the lack of appropriate new tools for control and eradication of these organisms. Although experimental models of protection show immunity to secondary challenge following drug-abbreviated primary infection, drug-induced clearance of helminths does not prevent rapid re-infection in human populations, and resistance to anthelmintic drugs has already emerged in veterinary use [2]. While vaccination would offer longer-term protection from infection, there are no currently available human anthelminthic vaccines, and the mechanisms of protective immunity on which new vaccines would depend have not been defined [3,4].

Helminth infection is, under natural conditions, near-ubiquitous and the mammalian immune system will have evolved specific mechanisms of activation and regulation to optimally respond to their challenge. Hence it is also likely that studying pathways of immunity to helminths will uncover new facets and properties of the immune system not apparent under conditions of infection with micro-organisms, and not necessarily predictable from our current knowledge of Type 2 activation in anti-helminth immunity [5,6].

The quest for anti-helminth vaccines began with live, radiation-attenuated organisms, which were effective in veterinary settings but unsuitable for human use [7]. Despite the successful translation of one veterinary vaccine to a molecular subunit formulation [8], in all other cases individual purified protein and recombinant vaccines have had modest effects, reducing worm loads without inducing full sterilising immunity in vaccinated recipients. While human trials have been initiated with partially-protective anti-hookworm and schistosome vaccines [9], it would appear that greater insight into the cellular and mechanistic basis of anti-helminth immunity, alongside the identification of protective antigens, will be essential if we are to enhance the choice and efficacy of future vaccines for human use.

Many helminths, of both humans and animals, are known to be highly immunomodulatory, mediating their effects at least in part through their repertoire of soluble secreted proteins which target the host immune system [10]. This has been particularly well-demonstrated in the natural mouse helminth parasite, *Heligmosomoides polygyrus*, which successfully establishes long-term infections in many strains of laboratory mice [11,12]. Immunomodulatory properties characterized in the *H*. *polygyrus* Excretory/Secretory (HES) products mediate a series of immunosuppressive effects on dendritic cells [13], airway epithelial cells [14] and T cells [15,16], prolonging parasite survival in an immunologically hostile environment.

In this study, we reasoned that if parasite secretions were promoting infection, that their blockade should generate protective immunity in this model system. As we demonstrate below, by immunizing mice with secreted products in an immunogenic fashion, we were able to generate complete protection against challenge infection. Protective immunization elicited IgG1 antibodies reacting against homologues of human vaccine candidates, as well as to several new targets conserved in parasites of human and veterinary importance. However, whilst a strong antibody response was a prerequisite for protection it alone was insufficient, and IL-25 signalling was also required for the expulsion of adult worms. Using intra-vital imaging of the intestinal mucosa, we show that protection is associated with enhanced recruitment and extravasation of LysM^+^ myeloid cells. Thus we show below that the antigen-specific adaptive response drives and unleashes a cytokine-dependent innate effector response for parasite elimination.

## Results

### HES immunization generates early immunity against larval parasite challenge

**Fig 1:**
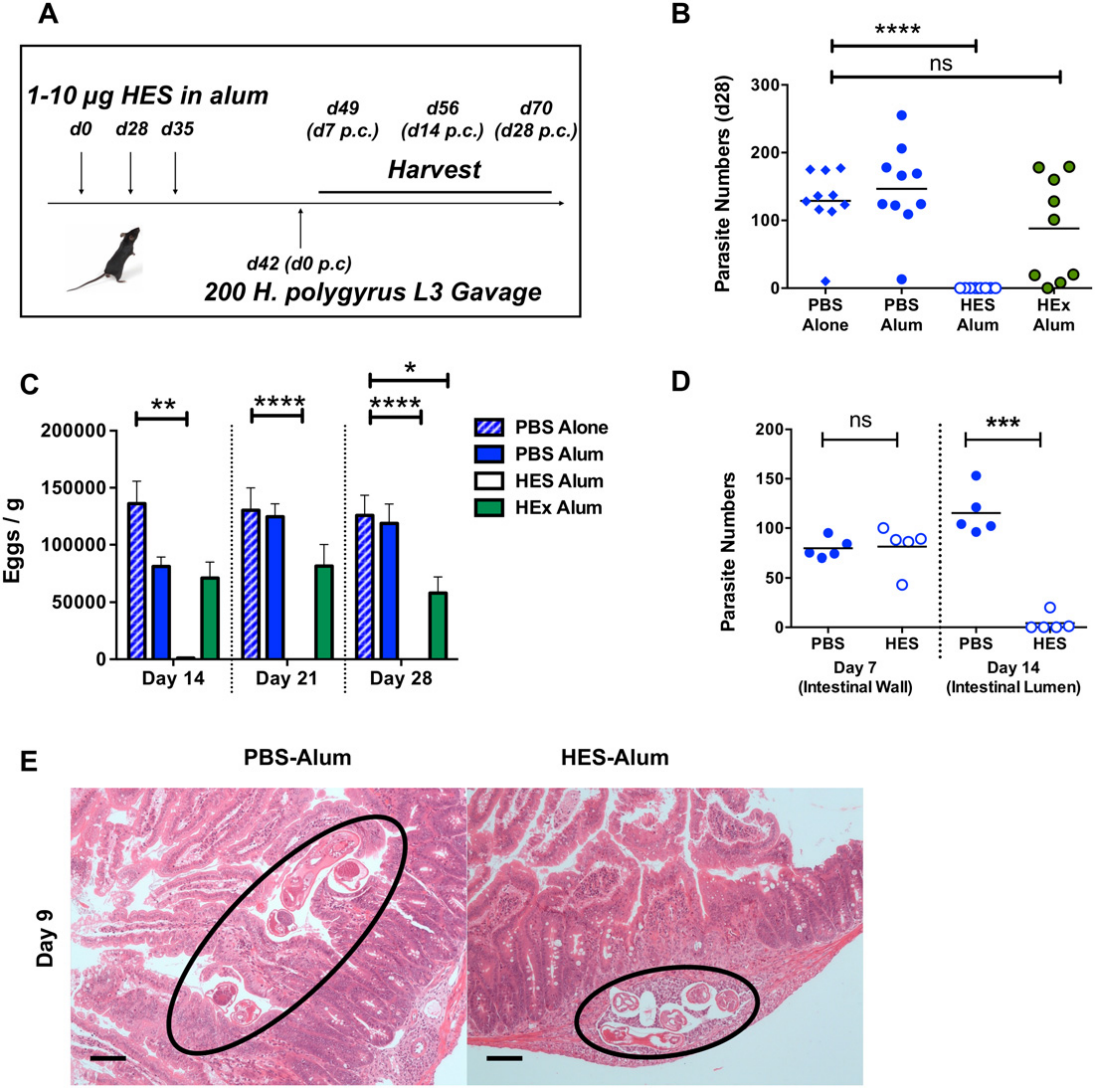
HES vaccination elicits sterile immunity to challenge and blocks parasite maturation. **A**. Schematic protocol for immunization. **B**. Parasite recoveries d 28 post-challenge following vaccination of C57BL/6 females with HES or somatic extract (HEx). Data shown are combined from two experiments each of 4–5 mice per group. Significance determined by ANOVA. **C**. Faecal egg counts (d 14, 21, 28 post-challenge) from (B). Significance determined by ANOVA. **D**. Parasite recoveries at d 7 (small intestinal wall) and 14 post-challenge (gut lumen). Representative of two independent experiments. Significance determined by *t*-test. **E**. H&E sections of duodenum in control and vaccinated animals at d9 post challenge; parasites in control animals are observed in gut lumen, but remain trapped in submucosa of vaccinated mice. Black circles indicate positions of parasite. Scale bar represents 100 μm.

We have previously reported that immunization with the secreted products of adult *H*. *polygyrus* (HES) confers potent immunity against challenge infection [17,18]. Using a standard alum adjuvant regimen (Fig. 1A), we first showed that while HES vaccination generates sterile immunity, as reflected by the complete absence of adult parasites at day 28 post-challenge, immunization with *H*. *polygyrus* somatic extract does not significantly reduce parasite numbers (Fig. 1B). Adult worm fecundity, as measured by fecal egg counts, is an earlier and more sensitive measure of immune attrition, by which HES vaccination is seen to elicit almost complete immunity by day 14, while the effects of somatic extract immunisation are considerably more modest (Fig. 1C).

The low level of eggs present in the feces of vaccinated mice at day 14 post-challenge (Fig. 1C) indicated that immunity was acting early in infection. To confirm this we established that at day 7 post-challenge, similar numbers of fourth-stage (L4) larvae are found in the intestinal wall of vaccinated and control mice, but by day 14 the immune animals had eliminated almost every helminth from the lumen (Fig. 1D). Notably, while worms in control mice had migrated to the gut lumen by day 9, many of those in vaccinated mice remained trapped in the gut wall (Fig. 1E). Thus immunity induced by HES vaccination is directed against the immature parasite, with worm immobilisation and expulsion taking place between the first and second weeks of infection. In this respect, the molecular vaccine reproduces previous observations of immunity against immature stages in mice rendered immune by prior infection and drug-induced clearance of parasites [19,20].

**Fig 2:**
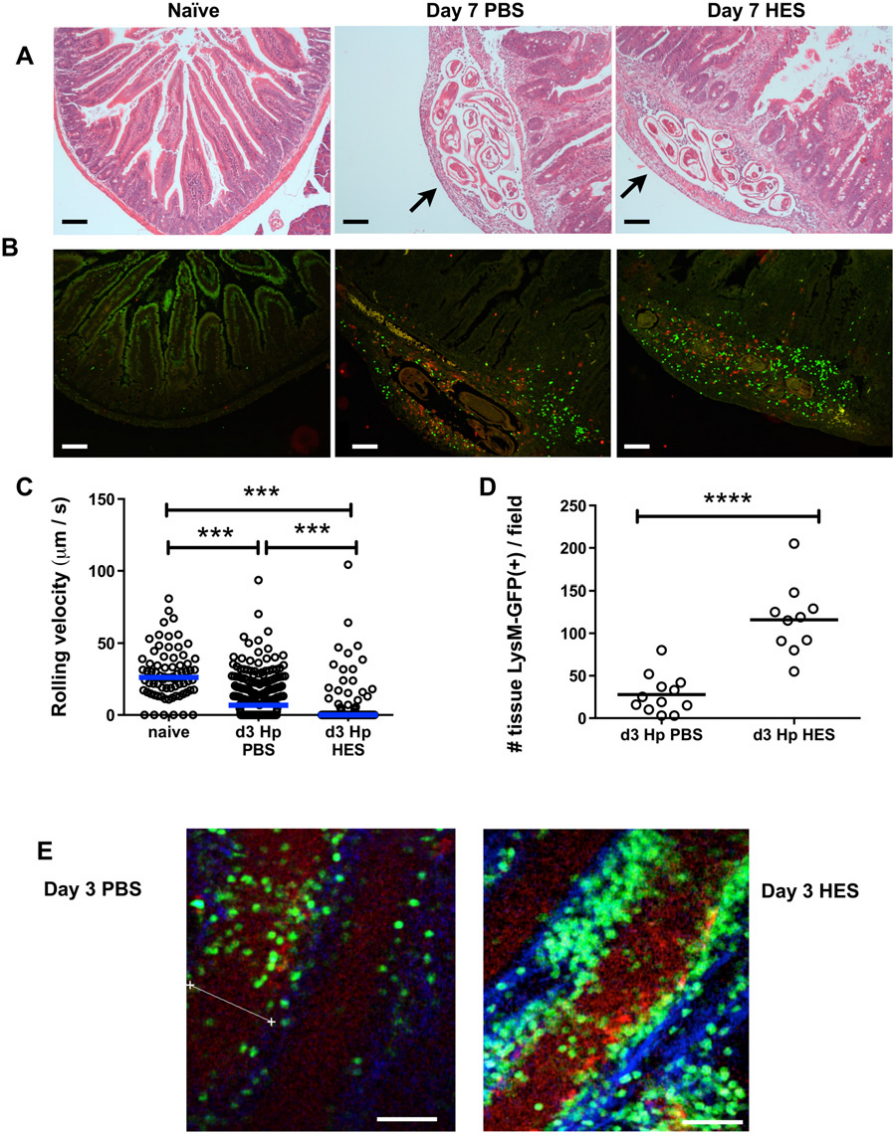
HES vaccination promotes extensive myeloid cell extravasation and accumulation around site of larval invasion. **A**. H&E staining of duodenal sections from naïve and d 7 post-challenge PBS control or HES-immunized C57BL/6 mice. Arrows indicate position of parasites. **B**. Immunohistology showing recruitment of CD11b^+^ (green) and Gr1^+^ (red) myeloid cells. Representative of 3–4 mice per group. Scale bar in A-B represents 100 μm. **C**. Rolling velocities of LysM-GFP^+^ cells along vessels imaged by two-photon microscopy of duodenum from the serosal aspect. Data accumulated from two mice per group, 3–7 vessels per mouse. Vessels were selected on basis of showing rolling behaviour. Significance determined by Kruskal-Wallis test. **D**. Tissue infiltration of LysM-GFP^+^ cells surrounding vessels in (C). Significance determined by *t*-test. **E, F**. Examples of two-photon microscopy from (D) showing tissue-infiltrating LysM-GFP^+^ cells in PBS- and HES-vaccinated mice. Scale bar represents 50μm. See also Supplemental Movie S1.

The early host response was further characterized immunohistologically at the inflammatory foci surrounding larvae in the intestinal sub-mucosa (Fig. 2A). By day 7, these inflammatory foci have attracted large numbers of CD11b^+^ and Gr1^+^ myeloid cells, which localise more intensely around the parasite in vaccinated mice (Fig. 2B). To gain a greater understanding of the dynamic events that occur *in vivo* following parasite challenge, we used intra-vital two-photon microscopy to image cells and larval parasites from the serosal side of the intact intestine in live LysM-GFP mice, which have labeled neutrophils, monocytes and macrophages [21,22]. Early in infection, at day 3 post-challenge, GFP^+^ leukocytes showed decreased rolling velocity, and increased arrest and extravasation in non-vaccinated mice compared to naive controls; however, GFP^+^ cell arrest (Fig. 2C) and tissue infiltration was greatly enhanced in vaccinated recipients, with a >4-fold increase in tissue infiltrating cell numbers (Fig. 2D-F and S1 Movie A-C). GFP^+^ infiltrating cells accumulated around parasite larvae which are still viable at day 5, but particularly in the immune mice are constrained and partly immobilised by the cellular infiltrate (S1D and E Movie).

Contrary to expectation, analysis of cell phenotypes in the intestinal tissue and draining mesenteric lymph nodes showed little difference between control and vaccinated animals, with similar levels of Th2 and Treg populations, although vaccinated mice have suppressed IFN-γ expression in the MLN (S1A-D Fig). In the lamina propria of infected mice, CD11b^+^Ly6G^+^ granulocytes, and both Ly6C^+^ and Ly6C^–^ CD11b^+^F4/80^int^ macrophages, showed expansion in all infected mice (S1E Fig), with a proportion of macrophages adopting an alternatively activated phenotype, characterised by expression of Ym1 and RELMα (S1F Fig). Notably, we observed higher levels of Ym1 transcript in total gut tissue from vaccinated mice by qPCR (S1F Fig).

### Immunity requires IgG antibodies but not activating FcR or complement

**Fig 3:**
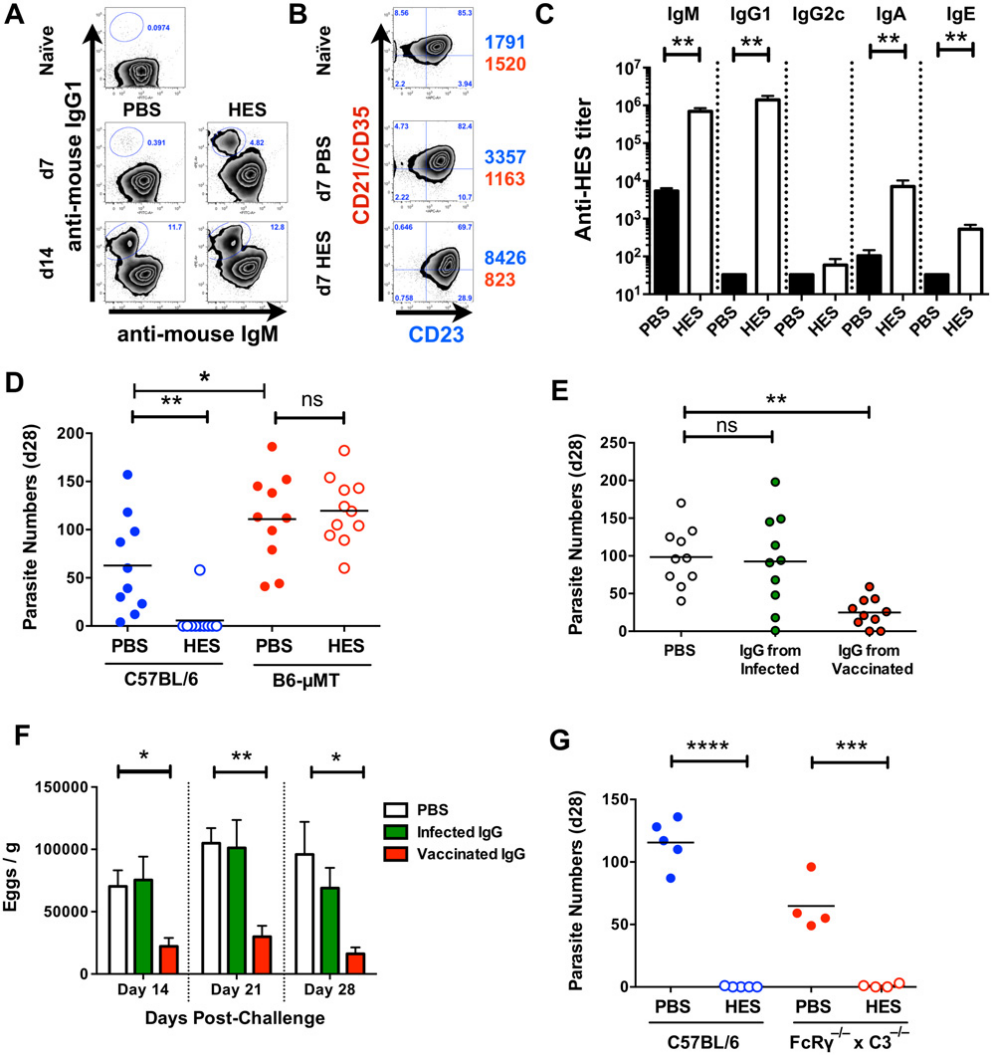
Immunity is dependent on cognate B cells and partially transferrable by antibody. **A**. Gated CD19^+^ MLN cell expression of cell surface IgM and IgG1 in naïve and d 7 and d 14 post-challenge PBS and HES B6 mice. **B**. Gated CD19^+^ MLN cell expression of cell surface CD21/CD35 (CR2/CR1) and CD23 (FcεRII) in naïve and d 7 post-challenge PBS and HES B6 mice. Numbers represent geoMFI of CD23 (blue) and CD21/CD35 (red) expression. A-B representative of two independent experiments with 3–5 mice per group. **C**. Pre-challenge anti-HES IgM, IgG1, IgG2c, IgA and IgE titers in PBS and HES vaccinated B6 mice with 5 mice per group. **D**. Day 28 post-challenge worm burdens from C57BL/6 and μMT mice following HES immunization or PBS control. Data pooled from two experiments. Significance determined by ANOVA as indicated. **E, F**. Day 28 post-challenge worm and fecal egg burdens (d 14, 21, 28) in naïve C57BL/6 mice receiving IgG from vaccinated or primary infected mice, or PBS, as detailed in materials and methods. Data are pooled from two experiments, with significance determined by ANOVA *Vs* C57BL/6 PBS. **G**. Day 21 post-challenge adult worm burdens in C57BL/6 and FcRγ^–/–^x C3^–/–^ mice following HES immunization or PBS control. Significance determined by *t*-test as indicated. See also S2 Fig.

Immunity to *H*. *polygyrus* generated by prior infection is known to be dependent on B cells, either or both through antibody and pro-Th2 cytokine production [19,23,24]. Vaccination induced marked phenotypic changes in MLN B cells with IgG1 class-switched CD19^+^ cells evident by day 7 post-challenge (Fig. 3A), alongside up-regulation of activation-associated CD23 (FcεRII), and reduced CD21/CD35 expression (Fig. 3B). Immunisation also evoked high titers of HES-specific IgG1 prior to challenge, as well as anti-HES IgA and E to a lesser degree (Fig. 3C). To assess whether B cells were required for immunity, we vaccinated wild-type C57BL/6 and congenic B cell-deficient μMT mice and found complete absence of immunity in the latter, as reflected in their higher worm and egg burdens (Fig. 3D, S2A Fig). To distinguish between humoral antibodies, and B cell-derived cytokines or signals, we also vaccinated MD4 mice with a fixed transgenic BCR for an unrelated protein, hen egg lysozyme (HEL). The inability of MD4 mice to develop protective immunity (S2B, C Fig) indicated that production of specific antibodies is critical for effective vaccination. Similar results were observed in CD40^–/–^ animals which were unable to class switch to parasite-specific IgG1 (S2D, E Fig), and which (as μMT mice; Fig. 3D) showed significantly elevated parasite numbers in response to primary infection (PBS control groups).

We next directly tested the protective potential of antibodies by passive transfer of purified IgG (predominantly IgG1, Fig. 3C) from the serum of HES-vaccinated animals into naïve recipients. Transfer resulted in significant reductions in adult worm (Fig. 3E), and egg (Fig. 3F) numbers, but not to the same extent as HES immunization. However, it was also noted that IgG transfer did not raise circulating anti-HES IgG1 titers in recipients to the same level as HES immunization (S2F Fig), and hence sterile immunity may require either extremely high antibody titers, or the activation of additional protective effector cell populations.

We next wished to see whether the protective antibody response was dependent on either activating FcR signalling and / or complement fixation. Vaccination of FcRγ^–/–^x C3^–/–^ double transgenic mice resulted in undiminished immunity (Fig. 3G) showing that neither FcR signalling nor complement activation is required for protection. This suggests that HES immunisation generates protective IgG1 antibodies that function by binding to, and directly neutralising the function of, essential parasite excretory / secretory (ES) molecules.

### Immunity requires IL-4 signaling, but is independent of eosinophils, mast cells, basophils and CCR2^+^ inflammatory monocytes

**Fig 4:**
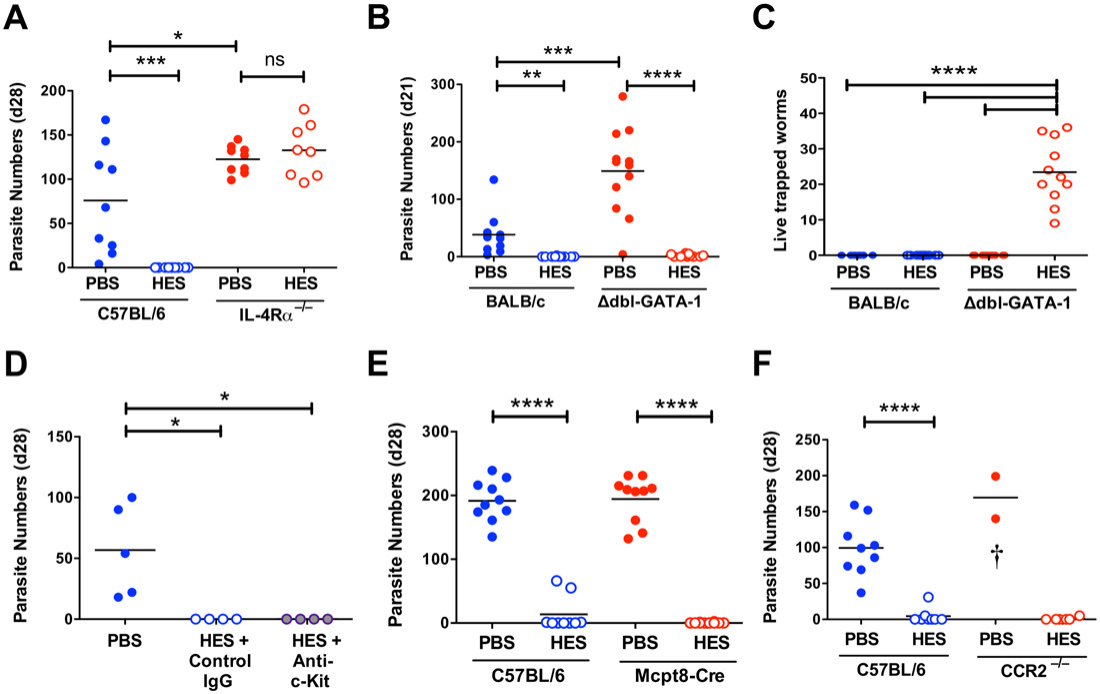
Vaccine-induced immunity requires IL-4R-mediated signaling but not eosinophils, mast cells, basophils or CCR2+ monocytes. **A-F**. Day 21–28 worm counts in control and vaccinated (A) C57BL/6 and IL-4Rα^–/–^ mice, (B-C) BALB/c and GATA-1 Δdbl mice, (D) ACK-2-treated and control C57BL/6 mice, (E) C57BL/6 4get/Mcpt8 cre and 4get controls and (F) CCR2^–/–^ and C57BL/6 controls. n.b. PBS/alum immunized CCR2^–/–^ mice died or were culled between 7–9 days post-challenge, with significant intestinal bleeding observed. Luminal worms in (A, B, D, E, and F), whereas counts in (C) represent live worms recovered from the intestinal wall at day 21 (i.e. presumed non-migratory parasites). Data in (A, B, C, E and F) pooled from 2 independent experiments. Significance in (A), (C) and (D) determined by ANOVA, in (B) by Kruskal-Wallis test, and (E-F) by unpaired *t*-test. See also S3 Fig.

To investigate cellular effector components that may be required to complement the protective effects of IgG1 antibodies, we first confirmed that immunity is fully dependent upon IL-4R signalling. Thus, vaccinated IL-4Rα^-/-^ mice fail to expel worms and actually incurred higher egg burdens than wild-type controls (Fig. 4A, S3A Fig), despite generating HES-specific IgG1 responses similar in titre to those seen in B6 primary infection (S3B Fig), and equivalent in specificity to immunised wild-type mice (S3C Fig).

We then investigated type 2 effector populations using gene-targeting or antibody-depletion approaches as appropriate. Because of the marked expansion in eosinophil numbers in MLNs of vaccinated mice (S3D Fig), we assessed immunity in two models of eosinophil deficiency, C57BL/6 IL-5^–/–^ mice and BALB/c Δdbl-GATA-1 mice (S3E-F Fig). Both eosinophil-deficient genotypes were fully protected from infection by HES vaccination (Fig. 4B and S3G Fig) and in both models anti-HES IgG1 titers were indistinguishable from wild-type mice (S3H, I Fig). Eosinophils have a role in killing parasites that are trapped in the intestinal wall of vaccinated mice, as a small but significant number of live worms (<40) were recovered from this site at day 21 post-challenge in vaccinated Δdbl-GATA-1 mice, but not BALB/c controls (Fig. 4C). Furthermore, PBS treated control Δdbl-GATA-1 mice had higher parasite burdens than their WT BALB/c counterparts, suggesting eosinophils have a role in the control of primary infection in this more resistant genotype (Fig. 4B).

Two other cells types thought to be important in Th2 mucosal immunity are mast cells and basophils. Mast cells gradually accumulate in the intestine of both control and vaccinated mice following challenge (S3J Fig). To focus on their potential role as effectors, rather than inducers, of type-2 immunity [25], we used the depleting anti-c-kit mAb (ACK-2) at the time of challenge. ACK treatment effectively depleted splenic mast cells, blocked serum mast cell protease mMCP-1 elevation and reduced intestinal levels (S3K-M Fig). Despite this, vaccine-induced immunity remained intact (Fig. 4D). The role of basophils was then assessed by immunization of basophil-deficient Mcpt8-Cre mice [26]. Again, HES vaccination rendered the mice fully resistant (Fig. 4E), indicating that immunity is intact in the absence of basophils as well as eosinophils and mast cells.

Similar genetic tools to selectively deplete other myeloid populations are less straightforward, and hence we employed clodronate depletion of phagocytes as previously described to impair immunity to secondary infection with *H*. *polygyrus* [27]. We found this treatment results in high haemorrhage-associated mortality (60–70%) in vaccinated and *H*. *polygyrus*-challenged mice, although the surviving mice remained immune. Because of the clear recruitment of Ly6C^+^ monocytes to the intestine following challenge (S1E Fig), and the requirement for IL-4R-dependent alternatively activated macrophages (AAMs) in resistance to secondary infection with *H*. *polygyrus* [27], we vaccinated and challenged CCR2^-/-^ mice. Despite the severe reduction of circulating monocytes in these mice [28], they showed full immunity (Fig. 4F). As such, whilst Ly6C^+^ monocyte influx may represent part of the normal intestinal response to a variety of inflammatory insults [29–31] it is not required for successful anti-helminth immunity. Instead, we observed significant mortality in infected control (but not vaccinated) CCR2^–/–^ mice, similar to that following clodronate treatment, revealing a key role for monocyte recruitment in either repairing parasite-induced damage as it migrates across the intestine, preventing bacterial translocation, or regulating the potentially pathogenic inflammatory response [31,32].

### Protection requires IL-25 signaling, independent of antibodies

**Fig 5:**
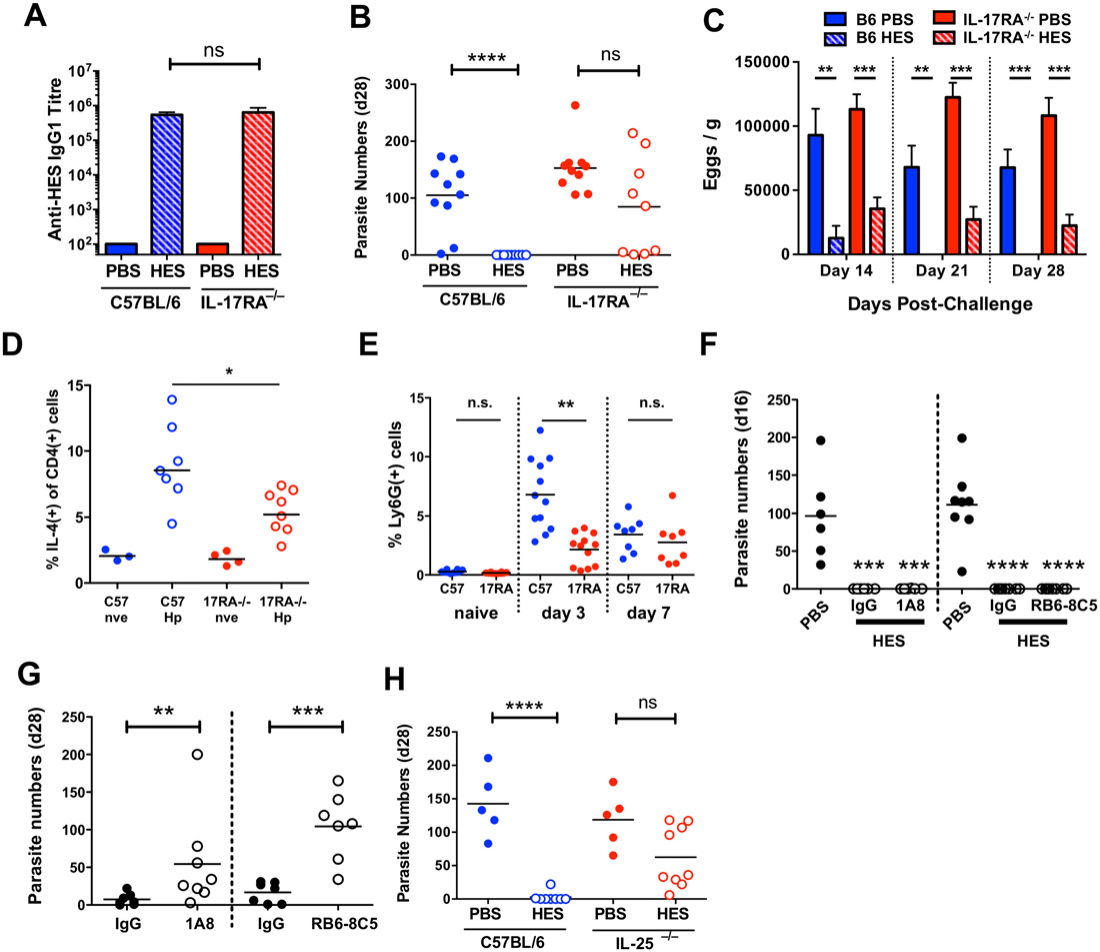
Vaccine-induced immunity requires IL-25. **A**. Anti-HES pre-challenge IgG1 titres in control and vaccinated C57BL/6 and IL-17RA-deficient mice. **B, C**. Day 28 worm counts and fecal egg burdens (d 14, 21 and 28) in control and vaccinated C57BL/6 and IL-17RA-deficient mice. **D**. Intracellular IL-4 production by naïve and d 7 post-challenge CD45^+^CD4^+^ lamina propria cells from C57BL/6 and IL-17RA^–/–^ mice. Pooled from two experiments. **E**. Proportion of lamina propria CD45^+^CD11b^+^Ly6G^+^ in naïve, d 3 and d 7 challenged C57BL/6 and IL-17RA^–/–^ mice. **F**. Adult worm burdens (d 16) in control and vaccinated C57BL/6 mice treated with anti-Ly6G (clone 1A8; left) or anti-Gr-1 (clone RB6-3C5; right) as detailed in materials and methods. **G**. Adult worm burdens (d 28) in primary infected BALB/c mice treated with anti-Ly6G, anti-Gr-1, or rat IgG control, as in (F). **H**. Day 28 worm counts in control and vaccinated C57BL/6 and IL-25-deficient mice. Significance in (A-E, G-H) determined by unpaired *t*-test or Mann-Whitney test, significance in (F) determined by ANOVA *Vs* PBS control. Data from A-G pooled from 2 independent experiments. See also S4 Fig.

Recent studies have shown a key role for IL-25 (IL-17E) in the induction of protective anti-helminth immunity [33], and for IL-17A in promoting helminth-induced inflammation [34]. To test if either IL-25 or IL-17A was important for vaccine-induced immunity, we first immunised mice deficient in IL-17RA (the shared receptor subunit for both IL-17 and IL-25), and found identical anti-HES IgG1 titers compared to WT animals (Fig. 5A), indicating neither IL-17 nor IL-25 signalling is required for alum adjuvant-induced antibody production. Surprisingly, HES immunisation failed in these mice (Fig. 5B), with all mice containing worms at day 28 (range 1–214), many having worm burdens similar to non-vaccinated controls. Vaccination did impair egg production, indicating that a qualitatively lower level of functional immunity was induced in the absence of IL-17/25 signalling (Fig. 5C).

IL-25 expands lineage-negative innate lymphoid ILC2s that promote Th2 differentiation [33], but intestinal ILC IL-5 production was unimpaired in IL-17RA^–/–^ mice (S4A Fig) and only a minor decrease in Th2 response was seen following challenge (Fig. 5D). It is important to note that in contrast to studies with the intestinal nematode *Nippostrongylus brasiliensis* [33,35,36] ILC2 expansion following *H*. *polygyrus* infection or challenge is relatively limited in susceptible wild-type C57BL/6 mice [12]. The muted induction of ILC2s by *H*. *polygyrus* implies that the parasite may have evolved effective means to suppress innate immune reactivity, such as that reported recently in the blockage of IL-33 release by its secreted products [14]. Instead, the major defect in IL-17RA^-/-^ mice was impaired early recruitment of CD11b^+^Ly6G^+^ cells to the intestine (Fig. 5E). Cell sorting revealed these cells to have an irregular ring nucleus (S4B Fig), as observed in “type 2” neutrophils elicited by *N*. *brasiliensis* infection [37]. Because IL-17 family cytokines (including both IL-17 and IL-25) stimulate neutrophilia, we determined whether neutrophil depletion would abolish vaccine-induced immunity to *H*. *polygyrus*. Using antibodies that deplete either Ly6G^+^ cells (with 1A8 antibody) or all Gr-1^+^ cells (with RB6-8C5 antibody to both Ly6C and Ly6G), we established that immunity following vaccination was unaffected by the loss of neutrophils (Fig. 5F). However, as reported previously [38], resistance to primary infection (assayed in the more resistant BALB/c strain) is reduced by neutrophil depletion, showing that these cells can contribute to protection in the setting of partial immunity (Fig. 5G).

Because the IL-17RA^–/–^ genotype does not distinguish between IL-17A and IL-25 signalling, we next immunised IL-25^–/–^ mice and found them to have a similar deficiency in vaccine-induced protection, indicating that IL-25 is an important factor in expressing protective immunity in this model, independent of parasite-specific antibodies, (Fig. 5H), and that ablated IL-25 signalling can account for the phenotype of the IL-17RA^–/–^ mouse, consistent with other data from our laboratory showing that antibody neutralisation of IL-17 does not influence susceptibility to *H*. *polygyrus* infection [39].

### HES contains multiple protective components

**Fig 6:**
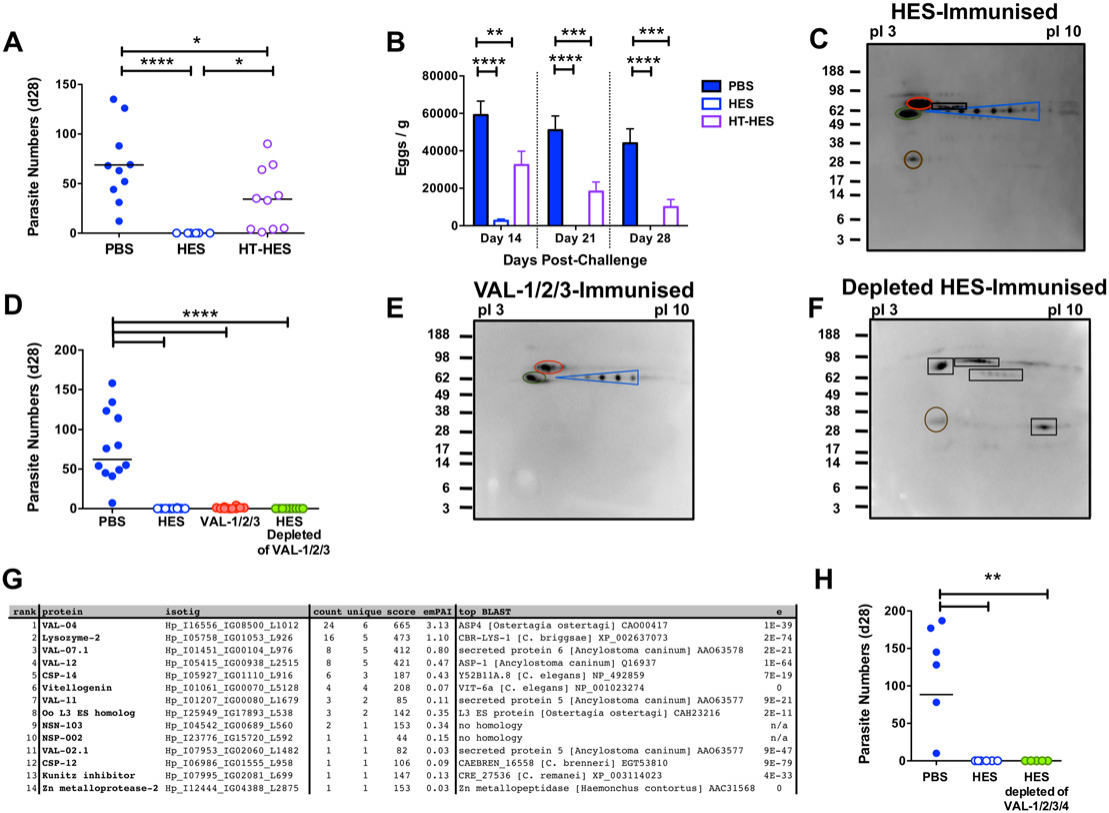
Identification of protective antigens following HES immunization. **A-B**. Day 28 post-challenge worm and fecal egg burdens (d 14, 21, 28) in C57BL/6 mice immunized with native or heat-treated HES. Data pooled from two experiments. Significance determined by ANOVA as indicated. **C**. Protective antibody targets revealed by immunoprecipitation of biotin-labeled HES by serum antibodies from immunized mice. Immunoprecipitated proteins were separated by pI (range 3–10) and molecular weights (indicated in kDa) and visualized with streptavidin HRP. Blue, red, green and brown circles represent VAL-1, 2, 3 and 4, respectively. Unknown antigens circled black. Sera pooled from 5 HES-vaccinated C57BL/6 mice pre-challenge and representative of two independent experiments. **D**. Day 28 post-challenge worm burdens from C57BL/6 mice immunized with a combination of VAL-1,-2 and -3, or with HES depleted of these 3 antigens. Data pooled from two experiments. Significance determined by ANOVA *Vs* PBS/alum. **E-F**. Immunoprecipitation of biotin-labeled HES antigens with vaccination sera as (C) from mice immunized with VAL-1/2/3 (E), or VAL-1/2/3 depleted HES (F). **G**. LC-MS/MS identification of antibody targets in mice immunized with VAL-1/2/3-depleted HES, performed on samples of unlabeled HES immunoprecipitated with serum antibodies from these mice. Proteins ranked according to spectral count (“count”). “Unique” represents number of unique peptide sequences in identification, “score” is Mascot score, and emPAI estimated abundance also shown. Highest scoring BLAST homolog with expect values indicated. **H**. Day 28 post-challenge worm burdens from C57BL/6 mice immunized with HES or HES depleted of VAL-1, -2, -3 and -4. See also S5 Fig.

To identify individual protective antigens within HES, we first established that immunity is elicited by heat-labile targets, rather than the abundant heat-stable glycans we recently identified [17]. Despite inducing similar titers of anti-HES IgG1 to native HES (S5A Fig), heat-denaturation markedly impaired the protective capacity of HES with all heat-treated HES immunised animals harboring between 1–90 adult parasites at day 28 (Fig. 6A). Nevertheless, heat-treated HES immunisation was still able to promote reductions in egg burdens (Fig. 6B). We then identified the dominant antigenic targets recognised by antibodies from vaccinated mice, using immunoprecipitation of native biotin-labeled HES, because key heat-sensitive epitopes are not detected by Western blot [17]. This analysis identified a restricted number of antigens, including known venom allergen-like (VAL) proteins, VAL-1, 2 and 3, and to a lesser extent VAL-4, as well as several additional spots (Fig. 6C).

Next, we purified native VAL-1, 2 and 3 with specific monoclonal antibodies [17], providing also a fraction of HES devoid of these three dominant antigens (S5B-F Fig). Mice immunised with HES or with a cocktail of VAL-1, 2 and 3 generated IgG1 antibodies against these proteins, whereas mice given VAL-depleted HES failed to do so, while generating antibodies to other specificities (S5G-J Fig). Immunisation with the three major VAL immunogens induced a high, if not quite complete, level of protection against challenge (Fig. 6D, S5K Fig). Although 5/10 mice harbored up to 4 adult parasites, with eggs detected in 7/10 mice at day 28, this represented a highly significant reduction compared to levels in control animals. In contrast, HES depleted of the three major VAL proteins induced robust sterile immunity to challenge infection (Fig. 6D), with no eggs detected from day 21 (S5K Fig). The antibody targets in VAL-1/2/3 depleted HES (Fig. 6E, F) were then identified by mass spectrometry of antigens immunoprecipitated by the appropriate vaccinated mouse sera as four additional members of the VAL superfamily (particularly VAL-4) as well as a lysozyme, two novel proteins and several others with homologs in other parasitic and non-parasitic nematodes (Fig. 6G). We then further depleted HES of VAL-4 (S5L, M Fig), in addition to VAL-1, 2, and 3, and found it to again fully protect against challenge (Fig. 6H). These results show that the protective components in HES include VAL proteins, as well as additional non-VAL proteins, which are likely to be protective in combination.

## Discussion

Together these studies show that immunization with secreted products from a gastrointestinal helminth induces long-lived sterile immunity against challenge infection through IgG1 antibodies acting in parallel with IL-4Rα- and IL-25-dependent effector cells. The ability of adult HES to drive immunity to immature stages is consistent with proteomic data demonstrating extensive antigen sharing between adult and larval secretomes, and the efficacy of ES from day 5 larvae in inducing full immunity [18]. Protective antibodies target both proteins of the VAL/ASP family that are known vaccine candidates [40], one of which induces natural IgE in exposed human populations [41], as well as non-VAL proteins with homologs in medically and veterinary important parasites. As antibodies induce protection in the absence of Fc receptor signalling, we suggest that they directly neutralise the function of parasite secretions.

Whilst antibodies reduce parasite fitness as reflected by egg production, they cannot alone lead to complete clearance of intestinal worms, which requires additional cytokine (IL-4Rα/IL-25)-stimulated cell population(s). We therefore hypothesise that protective antibodies neutralise parasite products that, in unvaccinated mice, are able to block the function of protective host innate cells. Such a model is consistent with greater myeloid cell recruitment to the intestine in vaccinated mice. Importantly, this suggests that successful vaccination against a mucosal pathogen may simply require the induction of sufficiently high titers of neutralising antibody to liberate host innate cells from parasite immunomodulation, allowing them to effect larval damage and killing.

While the ability of most HES-immunized mice to mount effective anti-larval immunity is striking, it is also notable that a small number of viable adult parasites survive to briefly produce eggs before they are also expelled. Two scenarios could explain this finding. First, parasites may be sufficiently damaged and stunted by immune attack in the tissues that their tenure in the luminal environment is short-lived. Secondly, there may be a distinct mechanism for expulsion of luminal parasites such as occurs to remove *N*. *brasiliensis*, whose tissue phase is within the lung. However, both tissue and luminal immunity appear to fully require activation through type 2 cytokines, and share many molecular and cellular pathways.

The dependence of helminth immunity on IL-4Rα signalling is a central paradigm established across many different systems [5,42] and suggests that indeed multiple Type 2 effector mechanisms are involved in elimination of *H*. *polygyrus*. However, we found that immunity in vaccinated mice was intact in the absence of various innate cell types reported to perform essential type-2 functions in other settings. For example, basophil-deficient Mcpt8Cre mice are more susceptible to primary infection with a related nematode, *Nippostrongylus brasiliensis* [26] and display impaired secondary immunity to *H*. *polygyrus* infection [43], yet are fully protected by HES vaccination. Likewise, mast cell-deficient W^sh^ mice are unable to clear primary *H*. *polygyrus* infection [25], but vaccine immunity is intact animals depleted of mast cells with ACK-2 antibody.

Eosinophils play intriguing roles in nematode infections, eliminating tissue-migrating larvae of some species [44–46], while in fact enhancing the survival of others [47]. In our system, the absence of eosinophils did incur higher primary levels of infection, but no deficit in immunity to challenge was observed other than greater viability of trapped larvae. Similarly, antibody depletion of neutrophils compromises resistance to primary infection but not vaccine-induced immunity to this parasite. So, whilst neutrophils may have some role in priming lung macrophages that promote early expulsion of *N*. *brasiliensis* following secondary challenge [37], they are not required for worm expulsion in the more chronic *H*. *polygyrus* model. An interesting observation is the expansion of CD11b^+^GR1^+^ subsets in the lamina propria, with phenotypes similar to myeloid-derived suppressor cells. However, they are unlikely to block immunity to the parasite as anti-Gr1 antibody compromises resistance to primary infection, and in the case of *N*. *brasiliensis* are able to promote expulsion of the luminal parasites [48,49].

Our finding that IL-25 is essential for vaccine-driven effector responses is somewhat surprising because it has been largely considered to be (alongside IL-33 and TSLP) a key inducer cytokine, acting at an early stage to drive ILC2s, mastocytosis and, in turn, Th2 immunity [50–55]. Whilst immunity to *H*. *polygyrus* is independent of TSLP [56] and IL-33 [57], IL-25 has a role in the partially protective response to primary infection [57]. Our data show that, whilst IL-25 is essential for the effector response that dislodges parasites from the intestine, it has little or no role in Th2 induction or the production of parasite-specific antibodies. This is most consistent with recent findings showing IL-25-deficiency does not to compromise the initiation of type 2 responsiveness following *N*. *brasiliensis* infection, but rather acts at a later stage to reduce immunity in both primary and challenge settings [58].

Interestingly, IL-25 is a key component in airway hypersensitivity, and antibody blockade of IL-25 in airway allergy was found to be most complete during the effector, rather than sensitization, phase [59]. Moreover, an IL-25-responsive myeloid cell was found to mediate allergic pathology in the lungs [60]. Although innate lymphoid cells, multipotent progenitor (mpp2) cells [61,62] and iNKT cells [63–65] are also responsive to IL-25, there are no data implicating any of these populations in the effector phase of anti-helminth immunity. Nevertheless, future studies will require lineage-specific deletions of the IL-25-specific receptor subunit (IL-17BR) to determine which populations are required to respond to this cytokine in the protective response to infection.

Resistance to secondary infection with *H*. *polygyrus*, following drug-induced clearance of primary parasites, is blocked by clodronate-loaded liposome depletion of phagocytes or pharmacological inhibition of Arginase-1, a key product of alternatively activated macrophages [27], while immunity to primary infection in genetically resistant mice is likewise compromised by clodronate administration [12]. In primary *H*. *polygyrus* infection, alternatively-activated macrophages adhere and immobilise tissue larvae [20], while transfer of macrophages (activated through IL-33) stimulates worm expulsion from chronically infected mice [66].

As has been noted elsewhere [67], the origin of intestinal macrophages during helminth-induced inflammation remains uncertain, and hence it is significant that immunity remains intact in CCR2-deficient mice. Monocyte egress from the bone marrow is impaired in these mice [28], and CCR2 ligation is also required for tissue entry, at least in Type 1 inflammatory settings [29]. Hence, immunity to *H*. *polygyrus* may be mediated by the coordinated recruitment of multiple (macrophage and non-macrophage) myeloid cell types utilising alternative receptors, but similarly driven through IL-4Rα and IL-25 to engage a similar effector gene program (including, for example, Arginase-1) to maximise immunity to the near-ubiquitous threat of gastrointestinal helminth parasites. Future work will be directed towards establishing if a suite of protective genes acting against helminth infection can be so defined, and if so the nature of their gene products and the range of cell phenotypes responsible for their production.

## Materials and Methods

### Mice, parasites, ES and extract preparation, immunization and antibody treatment

Mice were bred in house and kept in individually ventilated cages according to national guidelines. Transgenic strains were kindly provided as follows; C57BL/6 IL-17RA^–/–^ mice [68] by Prof B. Ryffel, Orleans France; C57BL/6 μMT, MD4, and CD40^-/-^ mice by Prof. D. Gray, Edinburgh, UK; C57BL/6 IL-4Rα^–/–^, IL-5^–/–^, and BALB/c GATA-1 Δ-dbl mice by Prof. J. Allen and Dr S. Babayan, Edinburgh, UK. C57BL/6 FcRγ^–/–^x C3^–/–^and CCR2^–/–^ mice were maintained in Lausanne, Switzerland, C57BL/6 4get x Mcpt8Cre mice [26] in Erlangen, Germany, C57BL/6 IL-25^–/–^ mice in the Malaghan Institute, New Zealand and C57BL/6 LysM-^+/gfp^ in Washington University in St. Louis, USA. HES, heat-denatured HES and adult somatic extract (“HEx”) was produced from adult *H*. *polygyrus bakeri* (originally provided by Professor JM Behnke, University of Nottingham, UK) as described elsewhere [17,69,70]. Mice were immunized essentially as before [17] with 5 μg HES in alum adjuvant i.p., then boosted with 1 μg in alum on days 28 and 35, before challenge with 200 *H*. *polygyrus* L3 generally 1–2 weeks later. Fecal egg counts were determined on days 14, 21 and 28 post-challenge, and intestinal adult worms counted as indicated. For passive immunization, total serum IgG from day 28 post-infection control or HES immunized C57BL/6 mice was purified using a AKTA prime fast protein liquid chromatography (LC) with a HiTrap protein G HP column, dialysed into PBS, and then injected into naïve recipient C57BL/6 mice (1mg) on days -1, 0, 1, 3, 5, 7, 10, 13, 15, 17, 20, 22, 24 and 27 post-challenge with *H*. *polygyrus*. The ACK-2 hybridoma was provided by Prof. Richard Grencis, and purified as above. Anti-Gr-1 clone RB6-8C5 was produced in-house at Bioceros, Netherlands. Anti-Ly6G clone 1A8 was purchased from Bio-X-Cell. Antibody depletion regimes were as follows; ACK-2 (1 mg i.p.) on days -1, 0, 1, 3 and 5 post-challenge, 1A8 (500 μg i.p.) and RB6-8C5 (250μg i.p.) on days -1, 0, 1, 3, 5 and 7 post-challenge. Control mice received rat IgG purified from serum as above.

### Ethics statement

All animal protocols adhered to the guidelines of the UK Home Office, complied with the Animals (Scientific Procedures) Act 1986, were approved by the University of Edinburgh Ethical Review Committee, and were performed under the authority of the UK Home Office Project Licence number 60/4105.

### Cell isolation and FACS

Mesenteric lymph node and lamina propria cells were isolated for antigen-specific restimulation, surface and intracellular staining, or polyclonal stimulation for intracellular cytokine staining, as will be described elsewhere [12]. For FACS analysis, single cell suspensions were stained with live/dead aqua (Invitrogen) as per manufacturer’s instructions, washed into FACS buffer (PBS with 0.5% BSA and 0.05% sodium azide), blocked with 500 μg/ml rat IgG (sigma) for 10 min on ice, then surface stained with the following fluorochrome-conjugated antibodies: CD45.2 (clone 104), CD11b (M170), F4/80 (BM8), Ly6C (HK1.4), Ly6G (1A8), CD3 (17A2), CD19 (6D5), CD8α (53–6.7), Gr-1 (RB6-8C5), CD11c (N418), MHCII (M5/114.15.2), CD4 (RM4-5), anti-mouse IgM (RMM-1), anti-mouse IgG1 (RMG1-1), CD23 (B3B4), CD24 (M1/69), CD117 (2B8); all from Biolegend. CD49b (DX5) and siglecF PE (E50-2440) from BD Pharmingen, and CD103 (M290) from eBioscience. Intracellular antibodies were: IL-4 (11B11), IL-17A (TC11-18H10.1), IFNγ(XMG1.2) from Biolegend; IL-5 (TRFK5), IL-13 (ebio13A), Foxp3 (FJK-16) from eBioscience. Samples were acquired using an LSR II or Canto flow cytometer (BD Bioscience), and analysed with flowjo software (Tree Star). Lamina propria CD11b^+^Ly6G^+^ cells were sorted using a FACS aria (BD).

### Cytokine and antibody ELISA

Serum antibody ELISA were performed as previously described [17] by coating with HES (1 μg/ml) or purified native VAL proteins (0.1 μg/ml), and detected using HRP-conjugated anti-mouse IgM, G1, G2a, A and E secondary antibodies (Southern Biotech). Controls included naïve mouse serum, day 28 primary infection sera and day 14 secondary infection sera. Gut homogenates were made in 1X cell lysis buffer (Cell Signaling) supplemented with PMSF protease inhibitor (Sigma) using a TissueLyzer (Qiagen). Serum and gut homogenate mouse mMCP-1 levels were determined with a commercially available ELISA kit (eBioscience).

### Quantitative PCR

RNA was TRIzol (Invitrogen) extracted from duodenum (approx. 5 mm) according to manufacturer’s instructions. RNA (1–2 μg) was reverse transcribed with M-MLV reverse transcriptase (Promega), and cDNA transcript levels measured by quantitative PCR on a Roche Lightcycler 480 II with SYBR Green (Roche) using primers described previously [71].

### Histology, immunohistochemistry, cytospins and two-photon microscopy

Transverse sections (5 μm) were cut from formalin-fixed paraffin-embedded duodenum and stained with either hemotoxylin and eosin or toluidine blue according to standard techniques. Pictures were taken using a DFC290 compound microscope and Application Suite software (both Leica). For antibody staining, paraffin sections were incubated with CD11b-FITC, Gr1-biotin/streptavidin APC. Intravital two-photon microscopy was carried out as previously described [22]. Briefly, mice were anesthetized with isofluorane, the peritoneal cavity opened, and duodenum (approximately 2–3 cm from the stomach) secured to a plastic coverslip with vetbond (3M). Non-targeted Q-dots (Qtracker 655nm, Invitrogen) were injected retro-orbitally to label the lumen of the blood vessels. Fluorescence was excited at 750 or 890 nm and images (~225 x 250 μm) were acquired from the serosal surface of intact intestine at 25 frames/second. Images were rendered and cells tracked using Imaris v7 (Bitplane, USA). Leukocyte recruitment was analysed as described in Kreisel *et al* [72].

### 2-D gel electrophoresis, western blotting, immunoprecipation and LC-MS

Identification of antibody targets in HES involved two separate approaches. First, for Fig. 6C, E and F, HES was biotin-labeled, immunoprecipitated with polyclonal sera from immunized mice, separated by 2-D gel electrophoresis and stained with HRP-conjugated Streptavidin as previously described [17]. Mice were immunized with specific VAL proteins purified from unlabeled HES by immunoprecipitation with Sepharose bead-conjugated mouse anti-VAL-1 (clone 5-S36), VAL-2 (clone 5-S2), VAL-3 mAb (clone 5-S1) and VAL-4 (clone 2–11). These mAb were generated from the spleens of secondary infected *H*. *polygyrus* mice and were conjugated as before [17]. In a second approach, shown in Fig. 6G to identify antigenic proteins by LC-MS, total serum Ig from uninfected C57BL/6 mice immunized with VAL-1, 2 and 3-depleted HES was isolated by ammonium sulfate precipitation and bead-conjugated as above. Immunoprecipitation of unlabeled total HES was carried out as above, and bound proteins were eluted and subjected to LC-MS/MS analysis as before [69]. Proteins were identified by comparison with a *H*. *polygyrus* transcriptomic assembly comprised of five different life-cycle stages (L3, day 3 larvae, day 5 larvae, adult and egg; Harcus *et al*, manuscript in preparation). MudPit scoring was used for LC-MS with a p<0.05 significance threshold, with single peptide hits more stringently filtered for expect values <0.01. False discovery rate for peptides at p<0.05 was 2.15%.

### Statistical analysis

Statistical analyses were carried out as indicated with Prism 6 (Graphpad Software Inc.). Normally distributed two-way comparisons used unpaired *t* tests, and multiple comparisons used one-way ANOVA, followed by Tukey’s test. If normality was not achieved, Mann–Whitney (for two-way comparisons) and Kruskal–Wallis tests (for multiple comparisons, followed by Dunn’s test) were used. P values of <0.05 were considered significant. * p<0.05, ** p<0.01, *** p<0.001, **** p<0.0001.

## Supporting Information

S1 Fig Characterisation of protective MLN and tissue responses post-challenge. (Related to Fig. 2) **A**. Naïve and d 7 MLN CD4^+^ intracellular IL-4 and IL-13. Graph shows IL-4/IL-13 double positive CD4^+^ cells from naïve, d 7 or d 14 MLN. **B**. As (A) showing MLN CD4^+^ cells intracellular IL-4 and IFNγin MLN. **C**. As (A) showing proportion of MLN CD4^+^ cells that are Foxp3^+^. **D**. As (A) showing proportion of MLN CD4^+^ Foxp3^+^ cells that are CD103^+^. **E**. Intestinal CD45^+^CD11b^+^ cell types in naïve and d 7 post-challenge control and HES-vaccinated mice; siglecF^+^, Ly6G^+^, F4/80^int^Ly6C^+^, F4/80^int^Ly6C^–^ indicated. **F**. qPCR for RELMα, RELMβ, Ym-1 and Arg-1 in duodenums from naïve (black) and d 7 post-challenge PBS controls (white) and HES immunized (black). Expression normalized to naïve levels. All data representative of two independent experiments. Significance determined by unpaired *t*-test as indicated.
(TIFF)

S2 Fig B cells, antibodies and immunity. (Related to Fig. 3). **A**. Faecal egg burdens (d 14, 21, 28) in control or vaccinated C57BL/6 and μMT mice. **B, C**. Adult worm (d 28) and faecal egg burdens (d 14, 21, 28) in control or vaccinated C57BL/6 and MD4 mice. **D**. Adult worm burdens (d 28) in control or vaccinated C57BL/6 and CD40^–/–^ mice. **E**. Pre-challenge anti-HES IgG1 titres in mice from (D). **F**. Anti-HES IgG1 in passive immunized mice following PBS injection (white), d 28 post-infection control IgG (green) or d 28 post-infection vaccine IgG (red) at d1 post-challenge (i.e. after 2x injections) or d28 (i.e. after 13x injections). Pre-challenge HES vaccine sera included for comparison. Significance in (A-F) determined by unpaired *t*-test as indicated. Representative of two experiments.
(TIFF)

S3 Fig Antibody-dependent and independent mechanisms of immunity. (Related to Fig. 4). **A, B**. Fecal egg counts (d 14, 21, 28) and pre-challenge anti-HES IgG1 titers in control and vaccinated C57BL/6 and IL-4Rα^–/–^ mice. Day 28 primary and d 14 secondary C57BL/6 infection sera included for comparison in (B). Pooled from two experiments. **C**. Immunoprecipitation of biotin-labeled HES antigens with pre-challenge vaccine sera from C57BL/6 and IL-4Rα^–/–^ mice. Mw markers and pI as indicated. **D**. MLN SiglecF expression from naïve, d7 and d14 post-challenge PBS and HES mice. Representative of two experiments. **E-F**. MLN SiglecF^+^ frequency in d 7 MLN from control and vaccinated C57BL/6 and IL-5^–/–^ (E) and BALB/c and Δdbl-GATA-1 (F) mice. **G**. Adult worm burdens (d 28) in control or vaccinated C57BL/6 and IL-5^–/–^ mice. Pooled from two experiments with 4–5 mice per group. **H-I**. Pre-challenge anti-HES IgG1 titers pre-challenge from C57BL/6 and IL-5^–/–^ mice (H) and BALB/c and Δdbl-GATA-1 (I) mice. Representative of two experiments. **J**. Toluidine blue^+^ MC cell numbers per 20 villus crypt units in duodenal sections from naïve and d 2, 5, 7 and 9 post-challenge PBS (blue) or HES immunized (red) mice. **K**. Splenic CD45.2^lo^ c-kit^+^ mast cells in naïve and infected C57BL/6 mice following ACK mAb treatment as detailed in material and methods. **L, M**. Serum (L) and gut tissue (M) mMCP-1 levels from (K). Significance determined by unpaired *t*-test (A, B, D, G-J) or ANOVA (K-M) as indicated.
(TIFF)

S4 Fig Type 2 immunity in IL-17RA^–/–^ mice. (Related to Fig. 5). **A**. Intracellular IL-5 and IL-17A production by live CD45^+^ lineage^–^ ICOS^+^ lamina propria cells from naïve and d 3 post-challenge C57BL/6 and IL-17RA^–/–^ mice. Lineage^–^ cells were gated as CD45.2^+^ lymphocytes that were CD3^–^CD4^–^CD8α^–^CD19^–^Gr-1^–^CD11b^–^CD11c^–^MHCII^–^F4/80^–^CD49b^–^. Representative of two experiments. **B**. Cytospins of FACS sorted CD11b^+^ Ly6G^+^ SiglecF^–^F4/80^–^ lamina propria cells from naïve and d 7 infected C57BL/6 duodenums. Scale bar represents 10 μm.
(TIFF)

S5 Fig Characterisation of protective antigens. (Related to Fig. 6). **A**. Pre-challenge anti-HES IgG1 titers in C57BL/6 mice immunized with PBS, native HES or heat-treated (HT) HES. Significance determined by unpaired *t*-test as indicated. **B-F**. 2-D silver stained gels of HES, VAL-1/2/3-depleted HES or native purified VAL-1, 2 and 3. VAL-1, 2 and 3 indicated in blue, red and green, respectively. Mw markers and pI as indicated. **G-J**. Pre-challenge anti native VAL-1 (G), 2 (H), 3 (I) and HES (J) IgG1 titers following immunization with PBS (black), HES (white), VAL-1/2/3-depleted HES (blue) and VAL-1/2/3 cocktail (red). **K**. Faecal egg burdens (d 14, 21, 28) in mice from (G-J). Significance determined by ANOVA *Vs* PBS/alum control. **L**. 2-D silver stained gel of native purified VAL-4 (brown circle) with Mw markers and pI as indicated. **M**. Pre-challenge anti native VAL-4 titers following immunization with PBS (black), HES (white) or VAL-1/2/3/4-depleted HES (blue). Data representative of two experiments (A, G-K) or multiple batches (B-F, L).
(TIFF)

S1 Movie LysM-GFP^+^ cell arrest and extravasation in infected tissue of immune mice. Intra-vital imaging of duodenal vasculature in uninfected LysM-GFP mice (**1 A**) or mice infected with *H*. *polygyrus* 3 days earlier (**1 B, C**), following immunisation with PBS-alum (**1 B**) or *H*. *polygyrus* ES antigens in alum (**1 C**). Green Fluorescent Protein (GFP) is expressed under the LysM promoter in neutrophils and monocytes, Q-tracker 655 (red) was injected intravenously to stain vascular contents, and the images were captured at 890 nm at which wavelength collagen fibrils emit second harmonic blue fluorescence. Two-photon imaging is real-time with scale bar representing 50 μm. Note most blood myeloid cells transit the vasculature of uninfected mice very rapidly without interacting with the vascular endothelium (**1 A**) Following infection cells show significant endothelial adhesion and crawling (**1 B**); however, extravasation is limited. In contrast, mice immunised against HES show extensive cell arrest and extravasation (**1 C**), indicating that immunity overcomes parasite inhibition of tissue inflammation. **Day 5 *H*. *polygyrus* larvae are enveloped by LysM-GFP** ^**+**^ **cells in the duodenal submucosa**. Intra-vital imaging of day 5 post-infection *H*. *polygyrus* nematode larvae encysted in the duodenal submucosa, in LysM-GFP mice. Images were captured at 750nm at which wavelength the nematode larvae autofluoresce in the blue range; intravascular Q-tracker 655 (red) is also visible. Two-photon imaging is real-time with scale bar representing 50 μm. Images compare control mice receiving PBS-alum injections (**D**) with HES-immunized animals (**E**). In immune mice, GFP^+^ myeloid cells show more extensive envelopment of the larvae, which are still alive at this point but are constrained and impaired by the cellular infiltrate. Also, note leakage of intravascular Q-tracker 655 into the parasite tissue niche, suggesting the larvae is exposed to vascular contents (including IgG1 antibodies).
(ZIP)
