# Supplementary figures and images for: Concerted Activity of IgG1 Antibodies and IL-4/IL-25-Dependent Effector Cells Trap Helminth Larvae in the Tissues following Vaccination with Defined Secreted Antigens, Providing Sterile Immunity to Challenge Infection
Source: PLoS Pathog. 2015 Mar 27;11(3):e1004676. doi: 10.1371/journal.ppat.1004676 (PMC4376884; doi:10.1371/journal.ppat.1004676)

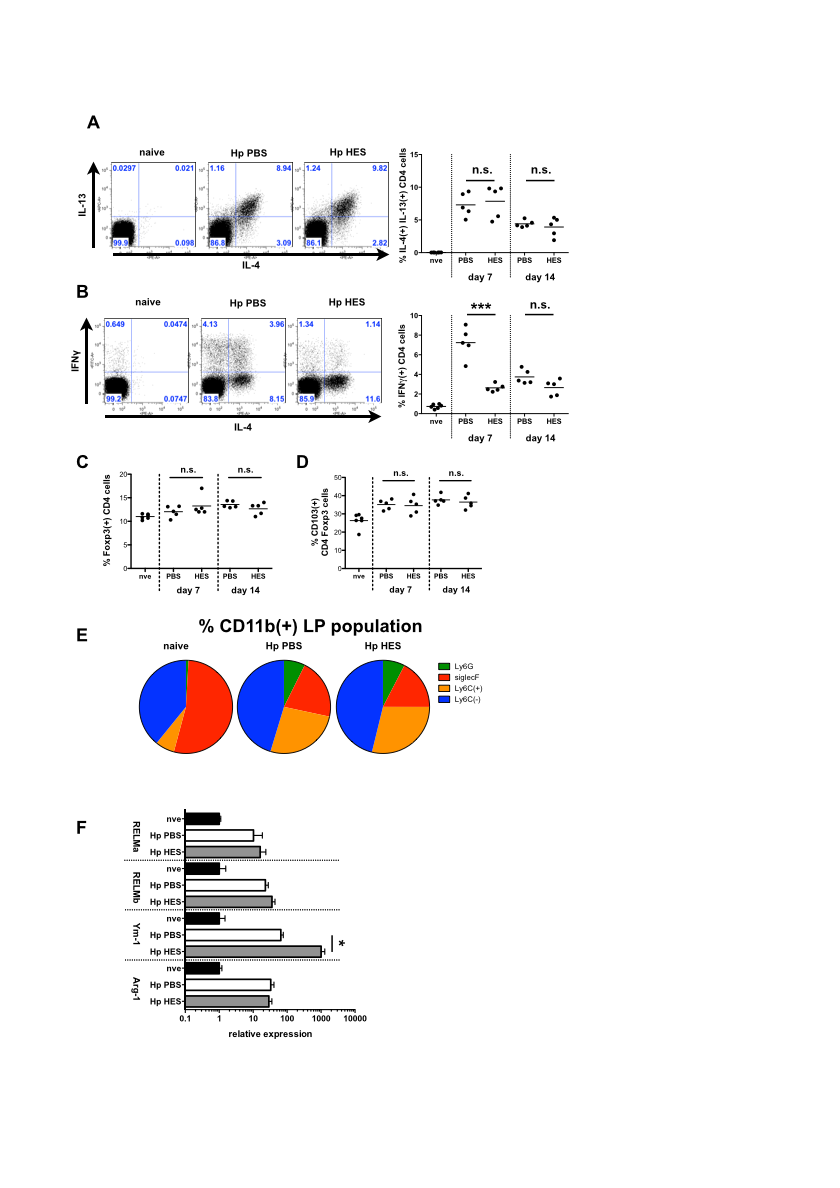

Supplement: S1 Fig — (Related to Fig. 2) A. Naïve and d 7 MLN CD4+ intracellular IL-4 and IL-13. Graph shows IL-4/IL-13 double positive CD4+ cells from naïve, d 7 or d 14 MLN. B. As (A) showing MLN CD4+ cells intracellular IL-4 and IFNγin MLN. C. As (A) showing proportion of MLN CD4+ cells that are Foxp3+. D. As (A) showing proportion of MLN CD4+ Foxp3+ cells that are CD103+. E. Intestinal CD45+CD11b+ cell types in naïve and d 7 post-challenge control and HES-vaccinated mice; siglecF+, Ly6G+, F4/80intLy6C+, F4/80intLy6C– indicated. F. qPCR for RELMα, RELMβ, Ym-1 and Arg-1 in duodenums from naïve (black) and d 7 post-challenge PBS controls (white) and HES immunized (black). Expression normalized to naïve levels. All data representative of two independent experiments. Significance determined by unpaired t-test as indicated. (TIFF) [file ppat.1004676.s001.tiff]

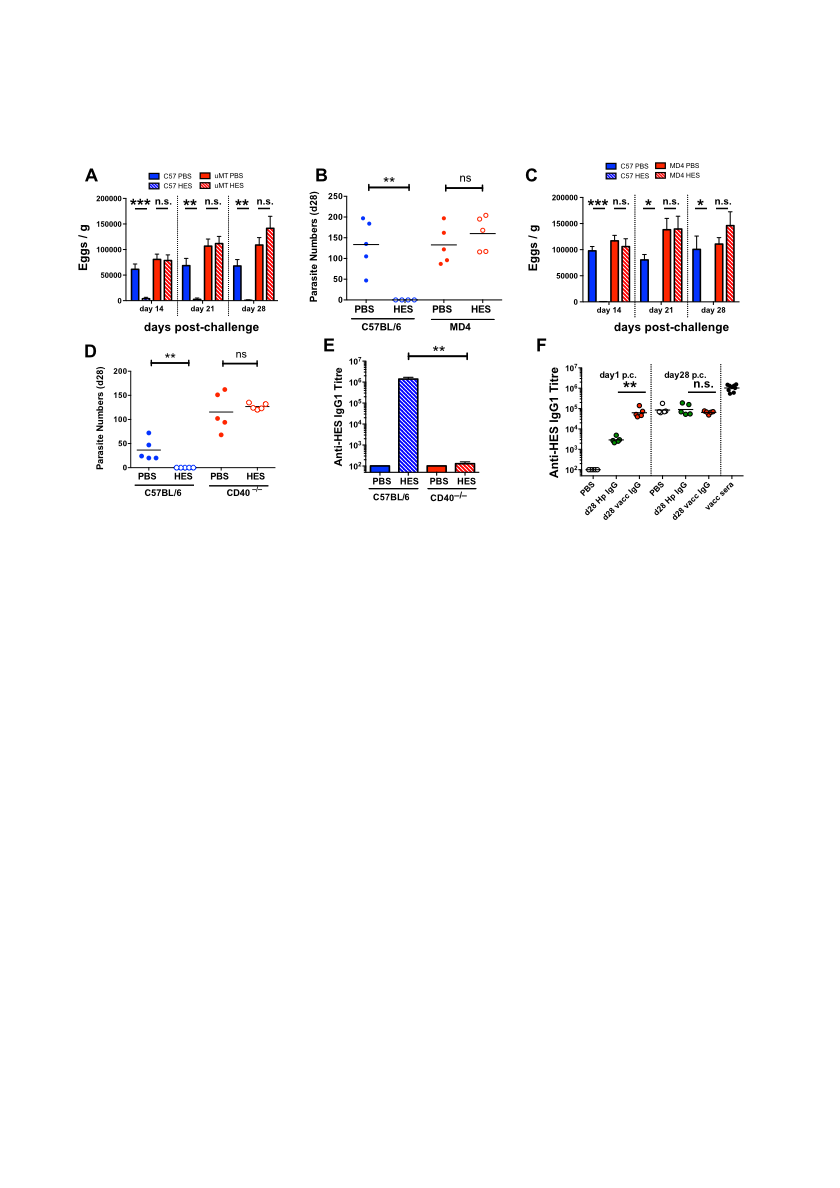

Supplement: S2 Fig — (Related to Fig. 3). A. Faecal egg burdens (d 14, 21, 28) in control or vaccinated C57BL/6 and μMT mice. B, C. Adult worm (d 28) and faecal egg burdens (d 14, 21, 28) in control or vaccinated C57BL/6 and MD4 mice. D. Adult worm burdens (d 28) in control or vaccinated C57BL/6 and CD40–/– mice. E. Pre-challenge anti-HES IgG1 titres in mice from (D). F. Anti-HES IgG1 in passive immunized mice following PBS injection (white), d 28 post-infection control IgG (green) or d 28 post-infection vaccine IgG (red) at d1 post-challenge (i.e. after 2x injections) or d28 (i.e. after 13x injections). Pre-challenge HES vaccine sera included for comparison. Significance in (A-F) determined by unpaired t-test as indicated. Representative of two experiments. (TIFF) [file ppat.1004676.s002.tiff]

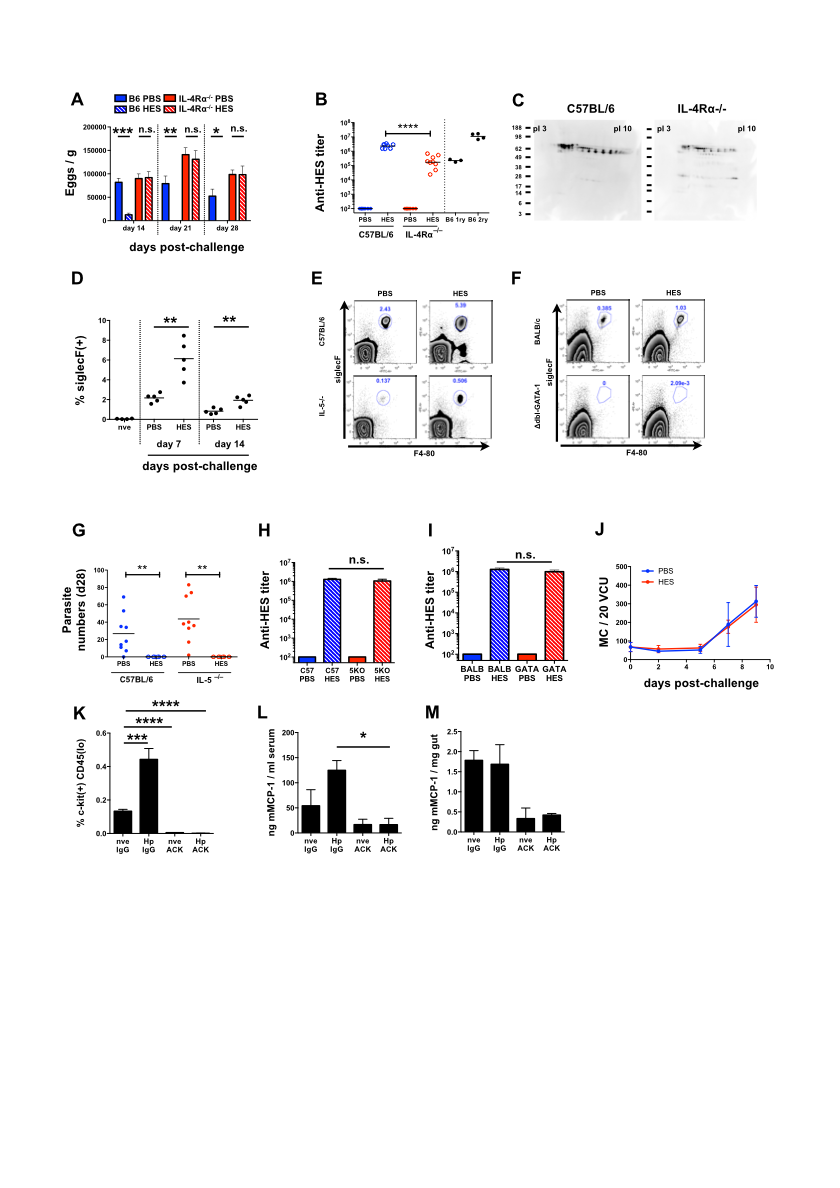

Supplement: S3 Fig — (Related to Fig. 4). A, B. Fecal egg counts (d 14, 21, 28) and pre-challenge anti-HES IgG1 titers in control and vaccinated C57BL/6 and IL-4Rα–/– mice. Day 28 primary and d 14 secondary C57BL/6 infection sera included for comparison in (B). Pooled from two experiments. C. Immunoprecipitation of biotin-labeled HES antigens with pre-challenge vaccine sera from C57BL/6 and IL-4Rα–/– mice. Mw markers and pI as indicated. D. MLN SiglecF expression from naïve, d7 and d14 post-challenge PBS and HES mice. Representative of two experiments. E-F. MLN SiglecF+ frequency in d 7 MLN from control and vaccinated C57BL/6 and IL-5–/– (E) and BALB/c and Δdbl-GATA-1 (F) mice. G. Adult worm burdens (d 28) in control or vaccinated C57BL/6 and IL-5–/– mice. Pooled from two experiments with 4–5 mice per group. H-I. Pre-challenge anti-HES IgG1 titers pre-challenge from C57BL/6 and IL-5–/– mice (H) and BALB/c and Δdbl-GATA-1 (I) mice. Representative of two experiments. J. Toluidine blue+ MC cell numbers per 20 villus crypt units in duodenal sections from naïve and d 2, 5, 7 and 9 post-challenge PBS (blue) or HES immunized (red) mice. K. Splenic CD45.2lo c-kit+ mast cells in naïve and infected C57BL/6 mice following ACK mAb treatment as detailed in material and methods. L, M. Serum (L) and gut tissue (M) mMCP-1 levels from (K). Significance determined by unpaired t-test (A, B, D, G-J) or ANOVA (K-M) as indicated. (TIFF) [file ppat.1004676.s003.tiff]

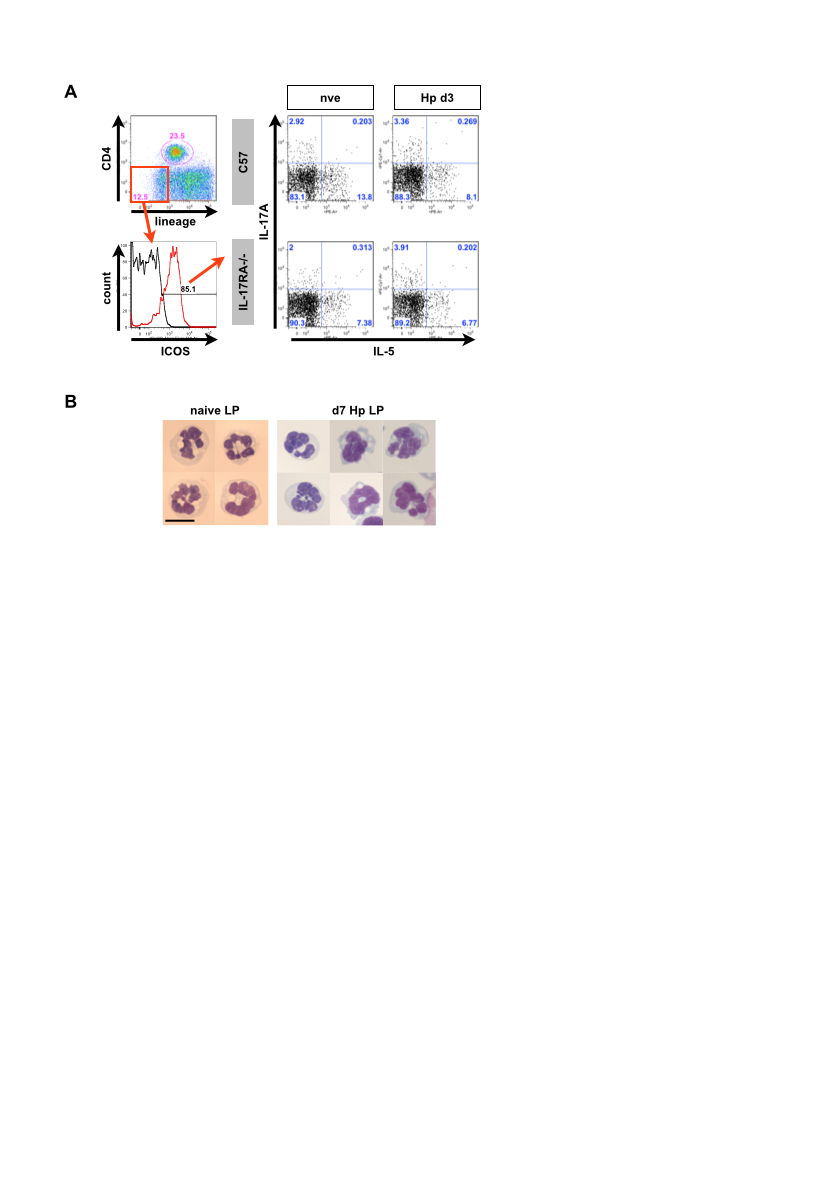

Supplement: S4 Fig — (Related to Fig. 5). A. Intracellular IL-5 and IL-17A production by live CD45+ lineage– ICOS+ lamina propria cells from naïve and d 3 post-challenge C57BL/6 and IL-17RA–/– mice. Lineage– cells were gated as CD45.2+ lymphocytes that were CD3–CD4–CD8α–CD19–Gr-1–CD11b–CD11c–MHCII–F4/80–CD49b–. Representative of two experiments. B. Cytospins of FACS sorted CD11b+ Ly6G+ SiglecF–F4/80– lamina propria cells from naïve and d 7 infected C57BL/6 duodenums. Scale bar represents 10 μm. (TIFF) [file ppat.1004676.s004.tiff]

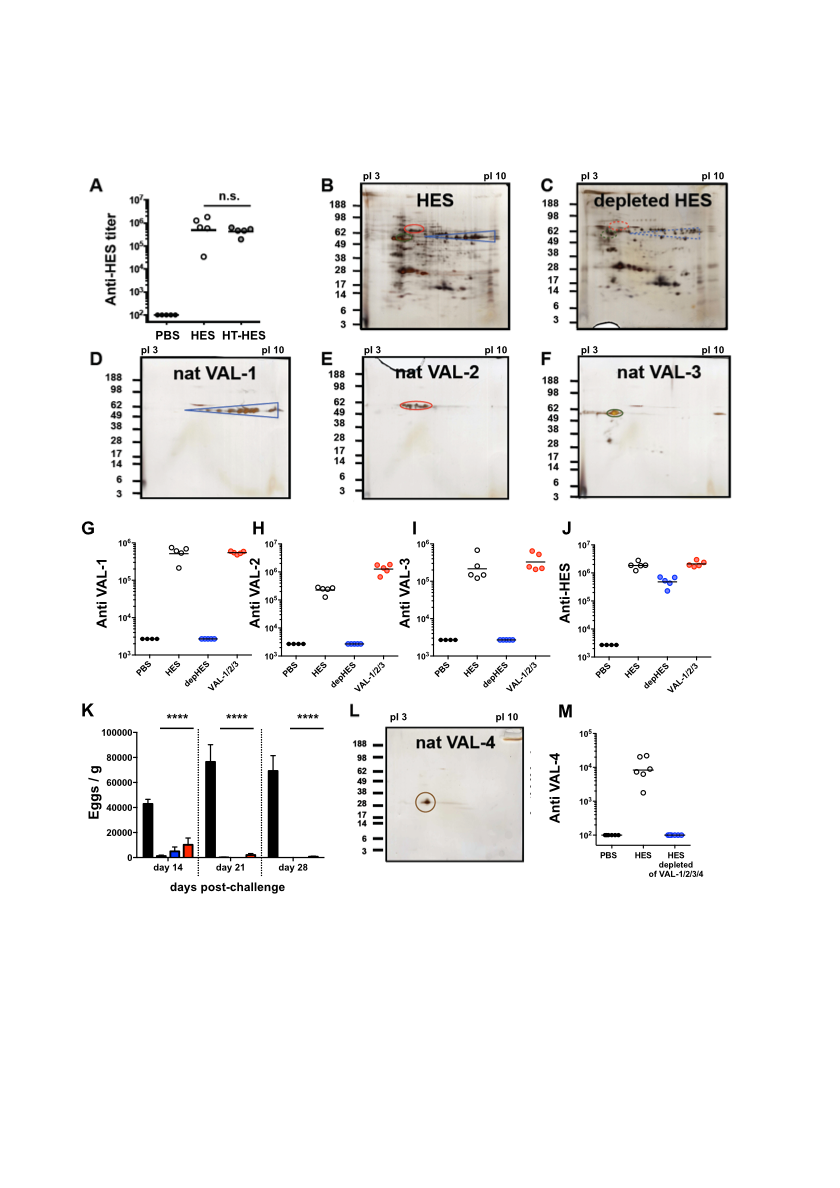

Supplement: S5 Fig — (Related to Fig. 6). A. Pre-challenge anti-HES IgG1 titers in C57BL/6 mice immunized with PBS, native HES or heat-treated (HT) HES. Significance determined by unpaired t-test as indicated. B-F. 2-D silver stained gels of HES, VAL-1/2/3-depleted HES or native purified VAL-1, 2 and 3. VAL-1, 2 and 3 indicated in blue, red and green, respectively. Mw markers and pI as indicated. G-J. Pre-challenge anti native VAL-1 (G), 2 (H), 3 (I) and HES (J) IgG1 titers following immunization with PBS (black), HES (white), VAL-1/2/3-depleted HES (blue) and VAL-1/2/3 cocktail (red). K. Faecal egg burdens (d 14, 21, 28) in mice from (G-J). Significance determined by ANOVA Vs PBS/alum control. L. 2-D silver stained gel of native purified VAL-4 (brown circle) with Mw markers and pI as indicated. M. Pre-challenge anti native VAL-4 titers following immunization with PBS (black), HES (white) or VAL-1/2/3/4-depleted HES (blue). Data representative of two experiments (A, G-K) or multiple batches (B-F, L). (TIFF) [file ppat.1004676.s005.tiff]
